# Reasons for Sports-Based Physical Activity Dropouts in University Students

**DOI:** 10.3390/ijerph18115721

**Published:** 2021-05-26

**Authors:** Iker Sáez, Josu Solabarrieta, Isabel Rubio

**Affiliations:** 1 Department of Physical Activity and Sport Science, Faculty of Psychology and Education, University of Deusto, 48007 Bilbao, Spain; irubio@deusto.es; 2 Department of Educational Innovation and Organization, Faculty of Psychology and Education, University of Deusto, 48007 Bilbao, Spain; josu.solabarrieta@deusto.es

**Keywords:** sport dropout, university students, physical activity, sedentary lifestyle

## Abstract

Despite extensive evidence reporting the numerous health benefits of physical activity, recent studies show that many people (60%) do not follow the recommendations to meet the accepted minimums of physical activity. Some of the main problems in today’s society are the high dropout rates (35%) and low adherence to the conditions for achieving the physical activity recommendations. The aim of the study is to analyze the reasons for dropout from sports that the participants particularly liked, to design a new scale of reasons as to why they dropped out based on several previously existing scales, and to study the dropout differences based on different variables. The sample consisted of 813 Vizcaya university students (61.6% women and 38.4% men), aged between 18 and 29 (M = 20.5; SD = 1.8). Frequency distributions, univariate descriptive analyses, and bivariate statistical analyses, such as t-test, analysis of variance, and correlations, were analyzed. The construct validity of the dropout reasons questionnaire was analyzed by combining an Exploratory Factor Analysis (n = 406) and a Confirmatory Factor Analysis (n = 407). The five-factor measurement model was appropriate and allowed to identify the factor rankings and its relation to some key variables. Lack of time (or dedication to other activities unrelated to physical activity) and fatigue seem to be the main factors for dropping out. The life changes produced at the time of entry and during university life seem to guide students to value other activities.

## 1. Introduction

For decades, participating in physical activities and sports has been considered a healthy habit [1,2]. Recent systematic reviews have shown that there are multiple psychological and social health benefits associated with sports participation for children, adolescents, and adults [3,4,5]. Engaging in physical activity and participating in youth sports is key in terms of development and in improving the mental, social, and physical health of young people [6,7]. Currently, there is established evidence on how increasing physical exercise promotes self-esteem, self-concept, and cognitive functioning, as well as alleviating depression levels in adolescents and adults [8]. However, the results obtained in different research related to the subject [9,10] indicate that a percentage of participants will completely or partially stop participating in sports-based physical activity. This situation is directly related to the quantity and quality of people’s lifestyles [11]. A sedentary lifestyle increases the rates of pathologies, such as obesity, cardiovascular diseases, diabetes, and different types of cancer, while also increasing the risk of mortality in the adult population [12,13].

Despite having broad evidence of the many health benefits of physical activity, recent surveys show that many people do not follow these recommendations [14,15,16]. In European countries, for example, recent estimates indicate that approximately 60% of citizens rarely or never participate in physical activities and sports and more than half of the population rarely or never engages in other regular physical activity (walking, climbing stairs, etc.) [17,18]. Worldwide, and particularly in the developed world, different daily or weekly measurements are used as a reliable measure for the level of physical activity in both adults and children [19,20,21]. Public health guidelines recommend that adults get a minimum of 150 min of moderate physical activity or 75 min of vigorous exercise per week [17,22]. In addition, and even more nowadays when multiple devices are accessible to the majority of the population in the developed world, Tudor-Locke et al. developed an index to analyze the number of steps taken as an indicator of physical activity levels [23,24,25]. Following the aforementioned indications: healthy adults who take 2500 steps/day are considered to operate at a baseline activity level; those who clock in 2500–4999 steps/day are described as having limited activity; the ones who complete 5000–7499 steps/day are considered low activity; adults taking 7500–9999 steps/day are classified as somewhat active; 10,000–12,499 steps/day are active; and 12,500/day are classified as highly active. This scale allows experts to objectively determine the level of physical activity of the population in general.

Today’s youth, especially university students, are considered a potentially vulnerable group due to the lifestyle changes they experience [26,27,28]. They often present risky behaviors, since they experience an increase in the number of hours sitting, an increase in alcohol or tobacco consumption, a decrease in the number of hours devoted to rest, loss of free time for outdoor recreational activities, leaving home and living alone, high exposure to stress, and greater access to inappropriate food-related habits [29,30]. All this constitutes a concerning scenario regarding the situation of young university students and is the reason why we focus on their situation, since during this period they create a bond with behavioral patterns that will be reproduced during adulthood [31].

Due to all the advantages that participating in physical activities and sports entails, the research has focused on the reasons why people participate; however, sports dropouts have increased as a research topic due to the high rates [32,33], with youth (approximately 18 years old) being a period of vital importance to study [34] since the regularity and dropout rate in adulthood occur at a lower degree. For example, in the US, during youth, 35% of those who participate in any sports program dropout annually [35]. Before beginning to analyze the concept of sports dropouts, it should be noted that in the research carried out the term “sports dropouts” is not clearly defined [36]. For this study, and based on Lindner et al. [37], we are going to understand dropouts as the result of quitting because the activity no longer satisfies their needs or because of exhaustion and, sometimes, because they quit a specific activity to participate in another activity or they end up quitting all activities.

This behavior is because the interests of young people change during the transition from youth to adulthood, and during this transition, the change from one sport to another or from participating to quitting [38] also takes place. Although the frequency of the rea-sons given for dropping out varies, based on the study, it is necessary to consider aspects related to the selection process, health (injuries or similar), chronological age, excessive pressure, lack of interest, problems with the coach, social environment (friends, family), media, lack of facilities [39,40,41], etc. In terms of differences between genders, the dropout rate is almost double between women and men [42,43]. However, if we add to this that, during youth, approximately [44] 61.3% of women compared to 46.7% of men do not meet the physical activity guidelines and that men are 20% more likely to participate in sports than women [45,46], it shows a situation in which dropout prevention is a real challenge [47].

The objective of this study is to analyze the reasons that led a sample of university students to dropout of sports that they particularly liked, to design a new scale of reasons as to why they dropped out based on several previously existing scales, and to study the dropout differences based on gender, age in which dropout occurred, the type of sports, and the differences between people who continue to participate in sports and those who stopped.

## 2. Materials and Methods

### 2.1. Subjects and Design

To conduct the study, 1309 questionnaires were administered among university students from different undergraduate studies who at the time were enrolled in universities in the Historical Territory of Vizcaya.

The participants were presented with the questionnaire as follows: “*Sometimes some people have to stop participating in physical activity or a sport that they especially like. Has this ever happened to you? Have you had to give up any sport that you liked a lot? If yes, please answer the following questions and, if not, skip the following questions. Next, focus on the activity you gave up. We are going to present you with a series of situations.*” Among the people who provided valid answers, 813 participants answered affirmatively to the question about dropping out and answered the questions presented below that make up the sample of this study. The ages of the participants ranged from 18 to 29 (M = 20.5; SD = 1.8). Out of the total, 501 (61.6%) were women and 312 (38.4%) were men. Before administering the questionnaire, all participants were informed of the objectives and nature of the questionnaire and completed an informed consent section.

### 2.2. Instruments

The questionnaire included questions about age, gender, and participating in some sports activity at the university stage. In addition, the participants were consulted about the situations in which people have to stop participating in a sport that they particularly liked. They were asked if they had to give up any sport that they liked very much. If yes, they were asked which sporting abandonment was the most frustrating for them, at what age the quitting happened, and to indicate the extent to which they dropped out due to any of the factors in the list, using a six-point Likert scale with each possible reason, rated from 0 (not at all) to 5 (a lot).

The sample represents a general population (university students) that participates in or participated in physical activity or different sports (organized or free) and that met the different dropout models: total or partial dropout or change of discipline. Usually, the reports found in the literature refer to athletes in specific sports (for example, swimmers, football players, etc.) who were either active or who dropped out. When the scientific literature available in Spanish is consulted, they are designed for and validated in populations associated with certain sports specialties, in many cases of high-level swimmers, football players, etc. Therefore, they report factors and items that may be less significant in non-specialized populations (for example, economic reward, titles and/or trophies, competitiveness, etc.).

The questionnaire was developed based on several studies on this topic. It explores the reasons behind dropouts [48] based on gender [49,50], sports participation [51], and age [52]. Before it was implemented, a group of university students contrasted it, and a pilot was implemented. With this, it was possible to improve the questionnaire by selecting the most relevant indicators and enhancing the wording of the questions.

### 2.3. Procedure

Before the questionnaire was administered, approval for the study was requested by the Ethics Committee of the University of Deusto, which was granted with the code “ETK-24/17–18.” The project considers the regulation for the protection of personal data (EU 2016/679) approved by the Commission and the Council of the EU in April 2016 related to the (i) informed consent procedure; (ii) access to personal data; (iii) the use of data for the public interest; and (iv) the responsibilities of the researchers responsible for the project.

Once the ethical suitability of the research was confirmed, further collaboration was requested from the University of Deusto and the University of the Basque Country to administer the questionnaire among their students. Once this collaboration was achieved, it was implemented, and the data were collected. The questionnaires were administered in the classrooms of the different departments during the students’ free time between classes. To guarantee accurate data collection and students’ understanding of the study’s nature, the main researcher of the study was present during the questionnaires’ administration. Before the questionnaire’s administration, all participants were informed of their willingness to participate and the confidentiality of the data collected. It is important to indicate that the participants did not receive any type of incentive. The data was collected between February and March 2018.

### 2.4. Statistical Analysis

The analyses consisted of frequency distributions, univariate descriptive analyses, and bivariate statistical analyses, such as t-test, analysis of variance, and correlations. The hypothesis testing used a significance level of 0.05. Statistical analyses were carried out using SPSS (v. 27) and Amos (v. 27).

The construct validity of the questionnaire regarding the reasons for dropping out was analyzed. Our questionnaire collects dimensions from various studies [53,54,55], which propose different factors that are sometimes juxtaposed. Therefore, we do not have a single theoretical model or a model for measuring the reasons for dropping out that is comprehensive enough. In the absence of having a foundation solid enough for the scale structure, we combined an Exploratory Factor Analysis and a Confirmatory Factor Analysis. We divided the sample into two halves, randomly assigning each case to one of these. In order to identify the underlying model in the responses in one part of the sample and check the model’s adjustment of the resulting measurement in the other, we successively carried out an Exploratory Factor Analysis with the first half (n = 406) and a Confirmatory Factor Analysis with the second (n = 407). The factors were extracted using Principal Axis Factoring and rotated using the Oblimin method. The Confirmatory Factor Analysis included calculations by bootstrapping, as well as the calculation of goodness-of-fit indexes, such as the Root Mean Square Error of Approximation (RMSEA), the Standardized Root Mean Square Residual (SRMR), and the Comparative Fit Index (CFI).

## 3. Results

Table 1 shows the extent to which the participants in the sample identified with each of the possible reasons for dropping out.

The most common reasons for dropping out are mainly lack of time and tiredness, followed by the preference for another sport, excessive pressure, and health reasons. They then select the problems with the coach, but the signs regarding problems interpreting the question are explained later. Excessive pressure or lack of company is mentioned less.

Before performing the Confirmatory Factor Analysis, we identified the questions whose correlation with the other items did not exceed the value of 0.3. In this way, five items were eliminated:

- 04 Health, injuries or illness;
- 06 Low academic performance;
- 11 It was expensive;
- 14 Lack of space or adequate facilities;
- 15 My father or mother encouraged me to dropout.

The Exploratory Factor Analysis used the Principal Axis Factoring main extraction method and requested an oblique rotation (Oblimin) to take into account possible correlations between factors (Table 2). Two other items showed cross-loadings and were eliminated: item 05 (after school sports, I did not do well in federated sports that divided its weight among the factors about sports-related bad results and lack of company), and item 02 (problems with the coach/way of training, relationship… sharing its weight among the factors related to poor results and excessive pressure). Finally, the Kaiser–Meyer–Olkin Measure of Sampling Adequacy was 0.764 and Bartlett’s Test of Sphericity’s *p*-value was 0.000 (Table 3).

Five factors explained 55.35% of the total variance. The first factor on bad sports results is made up of three items, the second factor on the amount of time required and tiredness contains three indicators, the third factor on excessive pressure includes three questions, the fourth factor on lack of company to participate in sports has two questions, and the fifth factor on lack of enjoyment also contains two questions.

**Figure 1:**
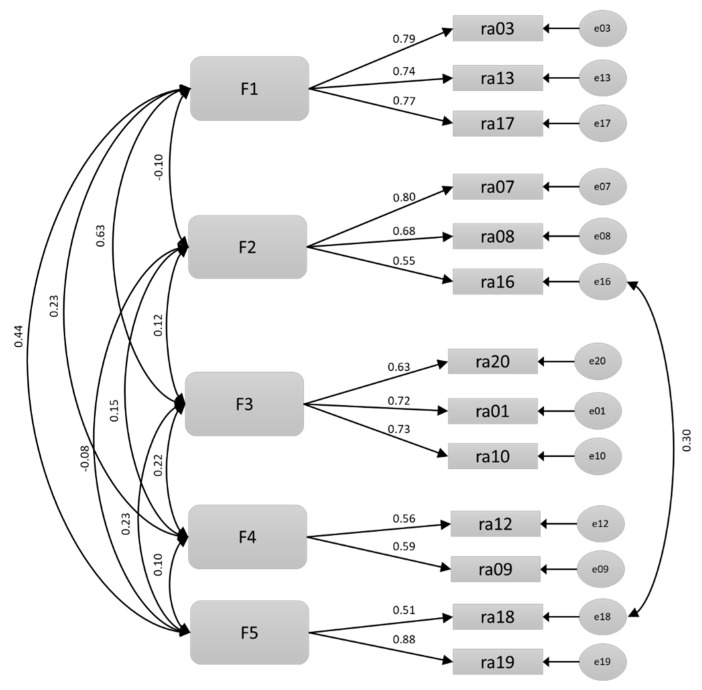
Measurement model of the scale of reasons for dropping out of sports.

This structure served as the basis for performing a Confirmatory Factor Analysis with the other half of the sample. The measurement model is shown in Figure 1.

The goodness-of-fit indexes (RMSEA = 0.060, SRMR = 0.058, CFI = 0.939) were appropriate.

The question on health, injuries, and illness was one of the most common, and the lack of correspondence with any latent variable may be due to its more directly observable nature, so it was included in the following analyses (Table 4).

The main difference between the genders is seen in Factor 5, lack of enjoyment, which includes dropping out due to preferring another sports activity; a moderately higher score is seen with men (d = 0.054). Four factors were found with statistically significant but small differences, but men indicate the factors of bad sports performance and lack of company more, while women are more prone to identify with excessive pressure, lack of time, and tiredness.

The people in the sample had dropped out of very diverse sports. We grouped the different types of sports and assigned each subject to one of the categories indicated in Table 5. Given the heterogeneity of the “Other Sports” category, the following comparative analyses do not take this category into account.

The lack of a company factor was the only one that showed no significant statistical differences based on the type of sports practice. The reference to bad sports performance is higher in collective sports such as handball, basketball, and football. The perception of excessive pressure as a dropout factor is somewhat higher in basketball, handball, and wrestling sports. Lack of time and tiredness are especially important in some predominantly individual sports, such as paddle and racket sports, followed by track and field sports, aquatic sports, aerobics, and wrestling sports. Lack of enjoyment is a factor that is indicated more in relation to dropping out of wrestling sports, handball, and aquatic sports. Health problems stand out in football, and to a lesser extent in track and field sports, wrestling sports, basketball, and handball (Table 6).

The age at which the people from the sample reported dropping out was between 7 and 24 (M = 15.3; SD = 3.0). Dropout age correlates significantly with lack of enjoyment and health problems (Table 7), but with opposite signs. Lack of enjoyment is less reported as a dropout factor when at an older age, while health reasons show an increasing incidence with age at the time of dropout.

From the people who reported the reasons why they had dropped out of a sport, 591 (72.7%) continued to participate in some sports at the university stage, while the remaining 222 (27.3%) did not (Table 8).

Within the dropout reasons, the only statistically significant difference between both groups was found with regard to lack of enjoyment, which was significantly lower in the group of people who had stopped participating in sports.

## 4. Discussion

Although multiple works [3,4,6] have highlighted the many benefits that participating in sports-based physical activity generates in people’s health, one of the main problems that physical activity and sports activity professionals encounter is the high dropout rates and low adherence present in young people. Sports dropout among active young people has been recognized as a global problem [56,57], and the results obtained in this study match the trends observed in the literature. Among the students surveyed, 69.4% reported having dropped out of some sports activity for some of the reasons previously listed, which resembles results such as those from the United States in which the dropout rate is around 70% [35], and those from Slovenia with a rate of about 75% [58]. According to a study carried out with university students in Spain, 6 out of 10 dropouts are in the age range of 19 to 23 years [59].

Factorial analysis allowed us to define the dropout reasons using the following factors: lack of time and tiredness, excessive pressure, lack of enjoyment, bad sports results, and lack of company. Added to this are health problems collected by a single question about health issues. With this analysis, we proved that personal factors (lack of time and tiredness, lack of enjoyment, and health and/or injuries) have the highest statistical weight, which is confirmed in other studies [32,55,60,61]. In addition, and in line with the literature [62,63,64], social factors (excessive pressure and lack of company) have the least statistical weight.

Lately, much attention has been given to identifying the reasons why young people stop participating in sports, and different models have been proposed that try to identify the different reasons that influence a young person’s decision to dropout [65]. In our study, as in other ones [54,66,67,68], we found that the lack of time to coordinate university, leisure, and sports is the most common reason for dropping out. As previously indicated in the introduction, the radical lifestyle change that occurs at the university stage makes structuring and coordinating physical activity with other activities a real problem [26,27,28]. We can connect dropout reason with having other activities at the same time. Tiredness is the second most common reason; it has been proven that, usually, a hard workout causes great fatigue that leads to dropping out [69,70]. However, in other studies [47,71], this argument was not shared since athletes understood that they had to train hard to achieve success. The reason with the next highest statistic weight is that related to pressure, in this sense as in other studies [72,73], when the sport level reached is high and when it focuses on competition (the ultimate goal is winning), there is a high psychological cost that can lead to dropping out. In addition, it is the continuous injuries and health problems that athletes face that make it really difficult to not dropout [74,75]. It is sufficiently demonstrated [76,77] that coaches play a very significant role in participants’ sports experiences, and athletes who enjoy a positive relationship with their coach have higher adherence rates compared to those who report more negative dynamics. In this study as well as in previous research [78,79], a high number of participants reported dropping out because of problems with the coach. Cross-loading the answers in the factor analysis shows the advantages of dividing the question in two in the future, first asking about problems with the coach related to poor results and then about problems related to the excessive pressure received from the coach [80,81]. High levels of fun and enjoyment are an adherence indicator, and it is not surprising that a lack of them is one of the main reasons for dropping out of sports; this fact is confirmed in various studies [80,82]. The final dropout reason with a significantly high level, is that participants’ self-perceived sports level along with their physical condition has a direct effect on the desire and motivation to not dropout of physical activity. This result has been contrasted in the same way in previous studies [83,84].

When analyzing gender-based dropout reasons for women, as recorded in other research [85,86], they have a significantly higher score than men on factors such as excessive pressure, lack of time, and tiredness. This is a result of the social responsibilities involving the female role to which academic responsibilities are added. For men, reasons like bad sports results, lack of company, or lack of enjoyment have higher scores than women, just as with previous studies [87,88,89]. Finally, no significant gender differences were found in reasons such as health and/or injuries, low academic performance, and the influence of parents, in the same line as other research [43,90].

Just as there are differences between dropout reasons between genders, the results obtained conclude, like other studies [33,91,92], differences in the number and dropout reasons between individual and collective sports. These differences can be the result of different situations (attribution of success or failure, inadequate equipment environment, perception of enjoyment, sports level, etc.) and different personal characteristics of the participants when selecting the type of sport.

In terms of dropout age, the older the age, the reasons for dropout are weighted as follows: lack of enjoyment, injuries, and low academic performance, most certainly due to the great changes that occur during aging of the studied population [38,40]. Furthermore, as participants get older, health problems increase due to the intensity of the activity, and the greater demand for physical condition and competition causes the lack of fun [93,94]. Additionally, in view of the results, those who dropout after moving from school sports to federated sports do so because in federated sports the perception seems to be a drop in results and the loss of socialization [95].

Finally, participants with sedentary behaviors (who completely dropped out of physical activity) would be expected to rate higher in the reason of “lack of enjoyment”, but the result is more complex, as in other studies [96,97]. It seems that active participants (who did not dropout or who dropped out partially) find enjoyment more important, and when they feel a lack of enjoyment, instead of dropping out they react more, looking for another alternate activities where they can have fun. This fact was confirmed in other studies [98,99].

## 5. Conclusions

The results highlight that sports-based physical activity dropout is a complex and multidimensional phenomenon. As explained in the introduction, it is vital to identify young people who completely dropout of sports and stop enjoying the benefits that physical activity provides in combating sedentary behaviors. In addition, it is important to understand the factors that cause partial dropouts or changes in discipline to avoid negative behaviors.

Based on these results, and on the target population of this research, sports managers of universities should take into account the factors obtained so that students have a maximum chance of participation and can continue with the activity. This can be achieved by offering activities compatible with students’ schedules, with an offer tailored to the needs, capacities, and interests of students. Sports policy should specifically prioritize sports retention and not just increase the total number of athletes.

This research provides a questionnaire to learn about the reasons for sports dropouts. The fact that it is brief and easy to implement promotes the feasibility of its use to learn the reasons for dropping out. In future versions of the questionnaire, it would be useful to differentiate the latent variables that reflect the internal constructs of the person and that requires at least three indicators each, from the most directly obvious variables (e.g., health problems), as well as differentiate the different factors within the same reference period in terms of lack of motivation (e.g., a coach who reports bad sports results versus a coach that puts excessive pressure on the players).

Lack of time and tiredness seems to be the main dropout factors. Changes in people’s vital priorities at the age at which they enter university seem to be aimed at a greater assessment of other activities. In this context, policies and programs should promote awareness of physical activity values, the merging of sports with ordinary university activities, and avoiding the loss of socializing values and leisure enjoyment that seems to occur during this transition period. During this period, competitive desire can prevail while weakening socialization and the leisure time of sports participation. Such interventions are essential to counteract the trends detected and need to be adapted specifically to the idiosyncrasies and context of this group of the population.

Different types of sports, and in particular collective versus individual sports, seem to be susceptible to different dropout factors, which may be helpful to decide how to prioritize the intervention of some dropout factors over others depending on each type of sport.

This study contributes to having more knowledge to improve strategies to reduce sedentary and unhealthy behaviors by studying the phenomenon of sports dropouts that needs to be understood over time in the general university population, and not only among people who participate in certain specialized sports intensely. It is a complex phenomenon to analyze, since sports-based physical activity occurs in different contexts and with different natures (competitive, recreational, free, organized, etc.) but its research is necessary due to the high number of dropouts that occur today. In the same way, we see it is necessary to continue with research in this area, validating instruments in specific populations and disciplines, but also in the general population.

Some limitations should be acknowledged and taken into account when interpreting the results. On the one hand, the study is retrospective, so it is susceptible to memory bias in the participants and to the difficulty of establishing causality. On the other hand, there could have been certain selection bias, although both genders, the academic year, universities, and the majority of sports practices with a greater number of participants and dropouts were well represented.

## Figures and Tables

**Table 1:** Means and standard deviations of the dropout questions.

	M	SD
07 Lack of time	2.54	2.03
08 I was tired from other activities (studies, etc.)	1.63	1.77
16 I had other activities at the same time	1.38	1.84
18 I prefer to participate in another sport	1.30	1.85
01 I experienced too much pressure	1.27	1.63
04 Health, injuries or illness	1.14	1.82
02 Problems with the coach (way of training, relationship)	1.04	1.67
17 I felt underappreciated or undervalued	0.91	1.50
10 It was too competitive	0.87	1.41
20 The training was too hard	0.79	1.36
03 My contributions to the group were scarce and I did not feel useful	0.66	1.25
09 My friends dropped out	0.65	1.26
12 Lack of company or not having someone to practice it with	0.63	1.28
19 It was boring	0.62	1.20
13 Bad results or poor sports performance	0.61	1.15
06 Low academic performance	0.55	1.16
11 It was expensive	0.43	1.06
15 My father or mother encouraged me to dropout	0.38	1.02
14 Lack of space or adequate facilities	0.36	1.00
05 After school sports, I was not doing well in federated sports	0.27	0.80

**Table 2:** EFA–total variance explained.

Factor	Initial Eigenvalues			Extraction Sums of Squared Loadings			Rotation Sums of Squared Loadings
	Total	% of Var.	Cum. %	Total	% of Var.	Cum. %	Total
1	3.66	28.14	28.14	3.24	24.96	24.96	2.63
2	2.07	15.95	44.09	1.63	12.52	37.48	1.54
3	1.52	11.71	55.80	1.06	8.16	45.64	2.35
4	1.18	9.06	64.86	0.74	5.67	51.31	1.45
5	0.91	6.97	71.84	0.53	4.04	55.35	1.48
6	0.61	4.72	76.56				
7	0.59	4.53	81.09				
8	0.54	4.12	85.21				
9	0.46	3.55	88.76				
10	0.42	3.21	91.96				
11	0.38	2.95	94.91				
12	0.37	2.85	97.76				
13	0.29	2.24	100.00				

**Table 3:** Exploratory factor analysis of dropout reasons questionnaire.

Label	Dropout Question	Factor				
		1	2	3	4	5
1 Bad sports results	03 My contributions to the group were scarce and I did not feel useful	0.98				
	13 Bad results or poor sports performance	0.65			0.13	
	17 I felt underappreciated or undervalued	0.57	−0.12	−0.22		
2 Lack of time and tiredness	07 Lack of time		0.84			
	08 I was tired from other activities (studies, etc.)		0.60	−0.19		
	16 I had other activities at the same time		0.56			
3 Excessive pressure	20 The training was too hard	−0.14		−0.84		
	01 I experienced too much pressure			−0.66		
	10 It was too competitive	0.18		−0.63		
4 Lack of company	12 Lack of company or not having someone to practice it with				0.75	
	09 My friends dropped out				0.58	
5 Lack of enjoyment	18 I preferred to participate in another sport					−0.80
	19 It was boring	0.17	−0.11	−0.13	0.23	−0.52

**Table 4:** Dropout reasons based on gender.

	M Men	M Women	Cohen’s	*t*-Test	*p*-Value
F1 Bad sports results	0.88	0.63	0.23	3.015	0.003
F2 Lack of time and tiredness	1.7	1.94	−0.16	−2.274	0.023
F3 Excessive pressure	0.86	1.07	−0.19	−2.624	0.009
F4 Lack of company	0.77	0.56	0.20	2.634	0.009
F5 Lack of enjoyment	1.4	0.69	0.54	7.294	0.000
fra04 Health, injuries, or illness	1.22	1.09	0.07	0.977	0.329

**Table 5:** Categorization of sports practices.

	Frequency	Percentage
Aquatic sports	110	13.5
Aerobics and others	55	6.8
Track and field sports	29	3.6
Basketball	133	16.4
Handball	31	3.8
Wrestling sports	35	4.3
Football	151	18.6
Paddle and racket sports	39	4.8
Other sports	230	28.3
Total	813	100.0

**Table 6:** Dropout reasons based on the type of sports practice.

	F1 Results	F2 Pressure	F3 Time	F4 Company	F5 Enjoyment	04 Health
Aquatic sports	0.48	1.03	2.18	0.61	1.14	0.86
Aerobics and others	0.55	0.83	1.94	0.47	0.65	0.89
Track and field sports	0.49	0.98	2.22	0.81	1.03	1.28
Basketball	1.07	1.41	1.46	0.67	0.90	1.2
Handball	1.17	1.21	1.30	0.87	0.68	1.1
Wrestling sports	0.79	1.14	1.83	0.76	1.31	1.26
Football	1.03	0.87	1.35	0.45	1.29	1.62
Paddle and racket sports	0.50	0.84	2.50	0.77	0.94	0.67
F	4.41	3.06	6.48	1.38	2.23	2.42
*p*-value	0.000	0.004	0.000	0.213	0.031	0.019
η2	0.05	0.04	0.07	0.02	0.03	0.03

**Table 7:** Correlations between dropout age and reasons.

	Pearson’s r	*p*-Value
F1 Bad sports results	−0.03	0.465
F2 Lack of time and tiredness	0.00	0.988
F3 Excessive pressure	0.01	0.807
F4 Lack of company	−0.01	0.771
F5 Lack of enjoyment	−0.29	0.000
ra04 Health, injuries, or illness	0.14	0.000

**Table 8:** Dropout reasons based on people who continue participating in sports and people who have stopped doing so.

	Mean Inactive	Mean Active	Cohen’s d	*t*-test	*p*-Value
F1 Bad sports results	0.77	0.71	0.05	0.76	0.449
F2 Lack of time and tiredness	1.75	1.88	−0.09	−1.16	0.245
F3 Excessive pressure	1.06	0.96	0.09	1.13	0.259
F4 Lack of company	0.62	0.65	−0.03	−0.34	0.738
F5 Lack of enjoyment	0.61	1.09	−0.36	−5.13	0.000
04 Health, injuries, or illness	1.2	1.12	0.04	0.57	0.570

## Data Availability

Data supporting reported results can be found by mailing authors.
